# Supplementary material for: Catecholaminergic modulation of the cost of cognitive control in healthy older adults
Source: PLoS One. 2020 Feb 21;15(2):e0229294. doi: 10.1371/journal.pone.0229294 (PMC7034873; doi:10.1371/journal.pone.0229294)
Supplement: S1 File — (DOCX) [file pone.0229294.s001.docx]

### Supplemental Material 1: Exclusion criteria

A potential subject who meets any of the following criteria will be excluded from participation in this study:

- Clinical dementia as measured by Mini Mental State Examination score < 24)
- Severe depression or anxiety as measured by HADS score > 11
- Estimated IQ < 85 (based on Nederlandse Leestest voor Volwassenen (NLV) -score)
- (History of) clinically significant psychiatric disorder
- (History of) clinically significant neurological disorder, such as brain infarct, Parkinson’s Disease, chronic migraine, Diabetes Mellitus
- First degree family history of schizophrenia, bipolar disorder or major depressive disorder
- Thyroid problems and low-protein diet
- Endocrine or metabolic disorders such as hepatic or renal problems
- Under treatment for
  - cardiac or vascular diseases and use medication for these conditions;
  - abnormal blood pressure < 90/60mmHg or > 160/90 mmHg (to be determined during the
  - intake session)
- Using medication that can interfere with tyrosine’s action; monoamine oxidase inhibitors and other antidepressants, sympathomimetic amines, and opioids
- General medical conditions, such as repetitive strain injury (RSI) or sensori-motor handicaps, blindness or colorblindness, as judged by the investigator
- (History of) abuse of drugs or alcohol
- Habitual smoking, i.e. more than a pack of cigarettes per week
- Participation, current or within the past twelve months, in a specific cognitive training study or previous study using the same paradigm as the current study
- Contra-indications for MRI: o Metal objects or fragments in the body that cannot be taken out
  - Active implants in the body
  - Using med ical plasters
  - Epilepsy
  - Previous head surgery
  - Claustrophobia
