# Supplementary material for: Catecholaminergic modulation of the cost of cognitive control in healthy older adults
Source: PLoS One. 2020 Feb 21;15(2):e0229294. doi: 10.1371/journal.pone.0229294 (PMC7034873; doi:10.1371/journal.pone.0229294)
Supplement: S2 File — (DOCX) [file pone.0229294.s002.docx]

### Supplemental Material 2: Mood / Blood pressure T2-T0

|  | **Measure** | **Screening** | **Placebo** | **Tyrosine** | **Drug effect** |
| --- | --- | --- | --- | --- | --- |
| **Mood**  (T2-T0) | Calmness | N/A | 0.0 (2.2) | -0.3 (1.5) | t(28) = 0.6, p = 0.566 |
|  | Contentedness | N/A | 0.2 (0.6) | 0.1 (1.0) | t(28) = 0.4, p = 0.689 |
|  | Alertness | N/A | 0.0 (1.4) | 0.4 (1.4) | t(28) = -1.2, p = 0.266 |
|  | Total | N/A | 0.0 (1.0) | 0.2 (1.1) | t(28) = -0.5, p = 0.599 |
| **Blood pressure**  (T2-T0) | Systolic | N/A | 7.2 (11.4) | 7.9 (10.8)* | t(27) = -0.3, p = 0.763 |
|  | Diastolic | N/A | 1.9 (4.4) | 3.9 (5.2)* | t(27) = -1.9, p = 0.062 |
|  | Heart rate | N/A | -2.0 (7.6) | -1.3 (6.3)* | t(27) = -0.6, p = 0.541 |

Data of mood and blood pressure assessments. Data represent mean (std) and results of paired-sample t-tests to assess intervention effects. Missing data points are marked by *.
