# Supplementary material for: Catecholaminergic modulation of the cost of cognitive control in healthy older adults
Source: PLoS One. 2020 Feb 21;15(2):e0229294. doi: 10.1371/journal.pone.0229294 (PMC7034873; doi:10.1371/journal.pone.0229294)
Supplement: S3 File — (DOCX) [file pone.0229294.s003.docx]

### Supplemental Material 3: Questionnaire N-back

For essay questions, please answer using your own words. There is no need to go into great detail, a few sentences should be enough for each question.

1. Please describe what you did to complete the CIRCLE task.
2. How difficult would you rate the CIRCLE task, on a scale of 1 to 10?

(very easy) 1 . . . 2 . . . 3 . . . 4 . . . 5 . . . 6 . . . 7 . . . 8 . . . 9 . . . 10 (very difficult)

1. How much effort did the CIRCLE task require, on a scale of 1 to 10?

(very little effort) 1 . . . 2 . . . 3 . . . 4 . . . 5 . . . 6 . . . 7 . . . 8 . . . 9 . . . 10 (very effortful)

1. Please describe what you did to complete the TRIANGLE task.
2. How difficult would you rate the TRIANGLE task, on a scale of 1 to 10?

(very easy) 1 . . . 2 . . . 3 . . . 4 . . . 5 . . . 6 . . . 7 . . . 8 . . . 9 . . . 10 (very difficult)

1. How much effort did the TRIANGLE task require, on a scale of 1 to 10?

(very little effort) 1 . . . 2 . . . 3 . . . 4 . . . 5 . . . 6 . . . 7 . . . 8 . . . 9 . . . 10 (very effortful)

1. How much effort did the TRIANGLE task require compared to the CIRCLE task?

The TRIANGLE task required … (check the most appropriate)

| much less effort | slightly less effort | about the Same | slightly more effort | much more effort |
| --- | --- | --- | --- | --- |
|  |  |  |  |  |

… compared to the CIRCLE task.

1. Please describe what you did to complete the SQUARE task.
2. How difficult would you rate the SQUARE task, on a scale of 1 to 10?

(very easy) 1 . . . 2 . . . 3 . . . 4 . . . 5 . . . 6 . . . 7 . . . 8 . . . 9 . . . 10 (very difficult)

1. How much effort did the SQUARE task require, on a scale of 1 to 10?

(very little effort) 1 . . . 2 . . . 3 . . . 4 . . . 5 . . . 6 . . . 7 . . . 8 . . . 9 . . . 10 (very effortful)

1. How much effort did the SQUARE task require compared to the CIRCLE task?

The SQUARE task required … (check the most appropriate)

| much less effort | slightly less effort | about the same | slightly more effort | much more effort |
| --- | --- | --- | --- | --- |
|  |  |  |  |  |

… compared to the CIRCLE task.

1. Please describe what you did to complete the DIAMOND task.
2. How difficult would you rate the DIAMOND task, on a scale of 1 to 10?

(very easy) 1 . . . 2 . . . 3 . . . 4 . . . 5 . . . 6 . . . 7 . . . 8 . . . 9 . . . 10 (very difficult)

1. How much effort did the DIAMOND task require, on a scale of 1 to 10?

(very little effort) 1 . . . 2 . . . 3 . . . 4 . . . 5 . . . 6 . . . 7 . . . 8 . . . 9 . . . 10 (very effortful)

1. How much effort did the DIAMOND task require compared to the CIRCLE task?

The DIAMOND task required … (check the most appropriate)

| much less effort | slightly less effort | about the same | slightly more effort | much more effort |
| --- | --- | --- | --- | --- |
|  |  |  |  |  |

… compared to the CIRCLE task.
