# Supplementary material for: Catecholaminergic modulation of the cost of cognitive control in healthy older adults
Source: PLoS One. 2020 Feb 21;15(2):e0229294. doi: 10.1371/journal.pone.0229294 (PMC7034873; doi:10.1371/journal.pone.0229294)
Supplement: S4 File — (DOCX) [file pone.0229294.s004.docx]

### Supplemental Material 4: Models

| **1. Choice** | |
| --- | --- |
| Model 1.1: | **SV** ~ Drug * Level * Amount + (1 + Drug * Level * Amount \| SubNo) |
| Model 1.2 | **SV** ~ Drug * Level * Amount * (**BIS-11 + Digit span**) + (1 + Drug * Level \| SubNo) |
| **2. Performance** | |
| Model 2.1: d’ | **d’** ~ Drug * Level + (1 + Drug * Level \| SubNo) |
| Model 2.2: RT | **log(RT)** ~ Drug * Level + (1 + Drug * Level \| SubNo) |
| Model 2.3: d’ | **d’** ~ Drug * Level * **(BIS-11 + Digit Span)** + (1 + Drug * Level \| SubNo) |
| Model 2.4: RT | **log(RT)** ~ Drug * Level * **(BIS-11 + Digit Span)** + (1 + Drug * Level \| SubNo) |
