# Supplementary material for: Catecholaminergic modulation of the cost of cognitive control in healthy older adults
Source: PLoS One. 2020 Feb 21;15(2):e0229294. doi: 10.1371/journal.pone.0229294 (PMC7034873; doi:10.1371/journal.pone.0229294)
Supplement: S5 File — (DOCX) [file pone.0229294.s005.docx]

**Supplemental Material 5: Need for cognition and SV**

Using this exact paradigm, the measure of SV has been shown to correlate positively with participants’ self-reported need for cognition scores (NCS). Including NCS in the choice model, unlike the earlier report, NCS scores did not relate to SV of the N-back task across drug (NCS effect: F(1, 27) = 0.41, p = 0.530).
