# Supplementary material for: Catecholaminergic modulation of the cost of cognitive control in healthy older adults
Source: PLoS One. 2020 Feb 21;15(2):e0229294. doi: 10.1371/journal.pone.0229294 (PMC7034873; doi:10.1371/journal.pone.0229294)
Supplement: S6 File — (DOCX) [file pone.0229294.s006.docx]

### Supplemental Material 6: Control analyses

We performed a number of control analyses using a model comparison approach, where we assessed whether the residual sum of squares was reduced when adding any of the following, perhaps confounding, factors to the SV model: order of drug administration, gender, age, and NLV scores (as a measure of verbal intelligence). Results of these control analyses confirm that none of the four models including these factors, one at a time, reduced the residual sum of squares significantly (all comparisons with basic model: X^2^(1) < 0.1, p > 0.90). ). In more recent projects, the order of catecholaminergic drug administration seems to interact with drug effects on subjective value of cognitive control (Hofmans et al., *in preparation*). Thus, here we assessed directly whether (i) our effect of interest (3.3) remains significant when taking into account order and (ii) whether effects interact with order. The analysis confirms that our effects of interest are not explained by order-effects: Drug x Level x BIS: F(1, 208) = 4.5, p = 0.034; Drug x Level x BIS x order = F(1, 208) = 0.1, p = 0.770.

Given that N-back data is available for 26 instead of 29 participants, we repeated the COGED analyses for the smaller sample as an additional control analysis: also in the reduced sample tyrosine significantly reduced the SV of the N-back task to a greater degree in more impulsive participants (drug x impulsivity: F(1, 22) = 4.60, p = 0.043) and trend-wise as a function of N-back level (drug x impulsivity x level: F(1, 22) = 3.90, p = 0.061).
