# Supplementary material for: Catecholaminergic modulation of the cost of cognitive control in healthy older adults
Source: PLoS One. 2020 Feb 21;15(2):e0229294. doi: 10.1371/journal.pone.0229294 (PMC7034873; doi:10.1371/journal.pone.0229294)
Supplement: S7 File — (DOCX) [file pone.0229294.s007.docx]

### Supplemental Material 7: Statistical effects of performance analyses

| **Effects** | **Performance** | | | |
| --- | --- | --- | --- | --- |
|  | Model 2.1: d’ | Model 2.2: RT | Model 2.3: d’ | Model 2.4: RT |
| **Level** | **F(1,25) = 129.5,**  **p < 0.001** | **F(1,25) = 21.0,**  **p < 0.001** | **F(1,22) = 147.6,**  **p < 0.001** | **F(1,22) = 18.5,**  **p < 0.001** |
| **Drug** | F(1,25) = 0.0,  p = 0.978 | F(1,25) = 0.9,  p = 0.342 | F(1,22) = 0.0,  p = 0.872 | F(1,22) = 1.9,  p = 0.178 |
| **Drug x Level** | F(1,25) = 0.3,  p = 0.596 | F(1,25) = 2.9,  p = 0.103 | F(1,22) = 0.4,  p = 0.512 | F(1,22) = 3.9,  p = 0.062 |
| **IMP** | N/A | N/A | **F(1,22) = 8.9,**  **p = 0.007** | F(1,22) = 0.0,  p = 0.851 |
| **Drug x IMP** | N/A | N/A | F(1,22) = 0.0,  p = 0.863 | F(1,22) = 1.5,  p = 0.236 |
| **Drug x Level x IMP** | N/A | N/A | F(1,22) = 1.0,  p = 0.323 | **F(1,22) = 4.9,**  **p = 0.038** |
| **Digit Span** | N/A | N/A | F(1,22) = 2.0,  p = 0.167 | F(1,22) = 0.0,  p = 0.885 |
| **Drug x Span** | N/A | N/A | F(1,22) = 0.1,  p = 0.822 | F(1,22) = 4.0,  p = 0.058 |
| **Drug x Level x Span** | N/A | N/A | F(1,22) = 0.4,  p = 0.544 | **F(1,22) = 6.0,**  **p = 0.023** |
