# Supplementary material for: Catecholaminergic modulation of the cost of cognitive control in healthy older adults
Source: PLoS One. 2020 Feb 21;15(2):e0229294. doi: 10.1371/journal.pone.0229294 (PMC7034873; doi:10.1371/journal.pone.0229294)
Supplement: S8 File — (DOCX) [file pone.0229294.s008.docx]

### Supplemental Material 8: Statistical effects of choice analyses.

| **Effects** | **Choices** | |
| --- | --- | --- |
|  | Model 1.1 | Model 1.2 |
| **Level** | **F(1,28) = 54.1, p < 0.001** | **F(1,25) = 51.5, p < 0.001** |
| **Drug** | F(1,28) = 0.2, p = 0.699 | F(1,25) = 0.1, p = 0.744 |
| **Drug x Level** | F(1,228) = 0.0, p = 0.912 | F(1,25) = 0.0, p = 0.853 |
| **Amt** | **F(1,28) = 4.8, p = 0.037** | **F(1,212) = 5.2, p = 0.023** |
| **Drug x Amt** | F(1,228) = 0.9, p = 0.338 | F(1,212) = 0.9, p = 0.342 |
| **Level x Amt** | F(1,228) = 2.8, p = 0.093 | F(1,212) = 2.9, p = 0.088 |
| **Drug x Amt x Level** | F(1,228) = 0.0, p = 0.912 | F(1,25) = 0.0, p = 0.853 |
| **IMP** | N/A | F(1,25) = 0.2, p = 0.673 |
| **Drug x IMP** | N/A | F(1,25) = 4.2 p = 0.051 |
| **Drug x Level x IMP** | N/A | **F(1,25) = 5.0, p = 0.034** |
| **Drug x Amt x IMP** | N/A | F(1,212) = 0.3, p = 0.605 |
| **Drug x Level x Amt x IMP** | N/A | F(1,212) = 0.1, p = 0.809 |
| **Digit Span** | N/A | F(1,25) = 5.6, p = 0.026 |
| **Drug x Span** | N/A | F(1,25) = 1.3, p = 0.268 |
| **Drug x Level x Span** | N/A | F(1,25) = 1.0, p = 0.320 |
| **Drug x Amt x Span** | N/A | F(1,212) = 0.2, p = 0.667 |
| **Drug x Level x Amt x Span** | N/A | F(1,212) = 1.6, p = 0.211 |
