# Supplementary material for: Catecholaminergic modulation of the cost of cognitive control in healthy older adults
Source: PLoS One. 2020 Feb 21;15(2):e0229294. doi: 10.1371/journal.pone.0229294 (PMC7034873; doi:10.1371/journal.pone.0229294)
Supplement: S9 File — Note that this model gives convergence-warnings due to model complexity. In-text, we describe results of Model 1.2 presented in S8 File. However, this table shows that our main conclusions on impulsivity-dependent tyrosine effects hold. (DOCX) [file pone.0229294.s009.docx]

**Supplemental Material 9:** Statistical effects of Model 1.2 now including ‘offer amount’ as random effect. Note that this model gives convergence-warnings due to model complexity. In-text, we describe results of Model 1.2 presented in Supplemental Material 8. However, this table shows that our main conclusions on impulsivity-dependent tyrosine effects hold.

| **Effects** | **Model 1.2 + random effect of ‘offer amount’** | |
| --- | --- | --- |
| **Drug x IMP** | F(1,25) = 4.2 p = 0.052 |  |
| **Drug x Level x IMP** | **F(1,25) = 4.7, p = 0.041** |  |
