# Supplementary material for: Catecholaminergic modulation of the cost of cognitive control in healthy older adults
Source: PLoS One. 2020 Feb 21;15(2):e0229294. doi: 10.1371/journal.pone.0229294 (PMC7034873; doi:10.1371/journal.pone.0229294)

### Supplemental Material 10: Choice trajectories

Choice-dependent offer adjustments of the low-effort task for the group average (top) and individual participants (bottom). The x-axis reflects the 5 trials of the titration procedure per cell (repeated for 3 effort levels indicated by shapes and 2 amount conditions indicated by the filling). The y-axis shows the offer amounts that can be gained when choosing the easy task. Based on participants’ choices, offers for the easy task (always N-back level 1) were adjusted. Note that the amount offered for the high-effort levels were not adjusted but fixed at €5 in the high amount condition (shapes unfilled) and at €2 in the low amount condition (shapes filled). The 3 effort-levels are presented by the according shapes (as in Figure 2C): triangle for the 2-back task, square for the 3-back task and diamond for the 4-back task.

Group figure: Whiskers depict standard errors of the means. For the 2-back task (triangle), the amount offered for the easy task increases as a function of trials because, on average, participants have a preference for the high reward/high effort option. For the 3- and 4-back task (squares and diamonds), the amount offered for the easy task decreases over time because particpants on average have a preference for the easy options.

Note that all shapes should be located at x = 1:5. For visualization purposes reducing the overlap in symbols, these are slightly shifted on the x-axis.

In sum, given the low frequency of correction for initial mistakes, the calibration of subjective values using two independent choice runs (low and high offer amount for the harder task) and the group plot indicating an overall consistent slope (lacking the sign of systematic initial mistakes), we believe that the current subjective values reflect participants’ preferences fairly well.


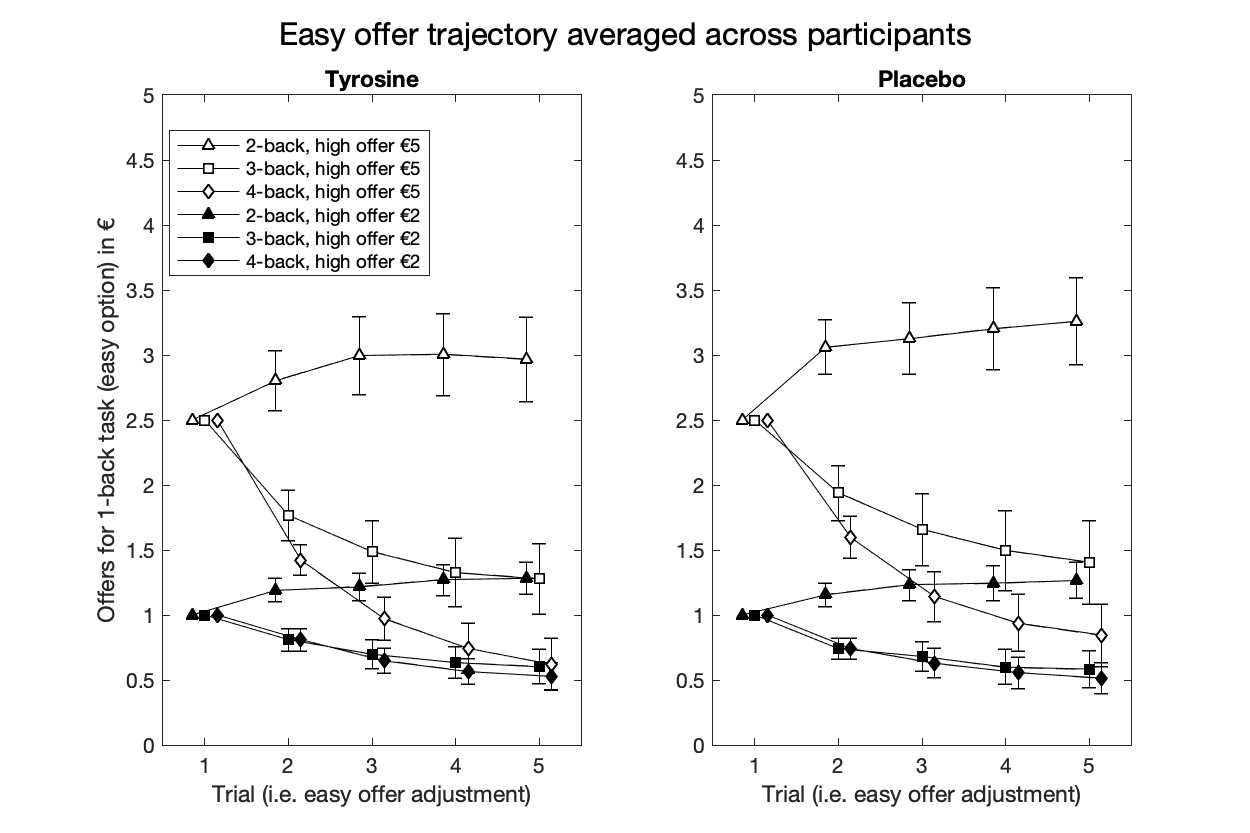


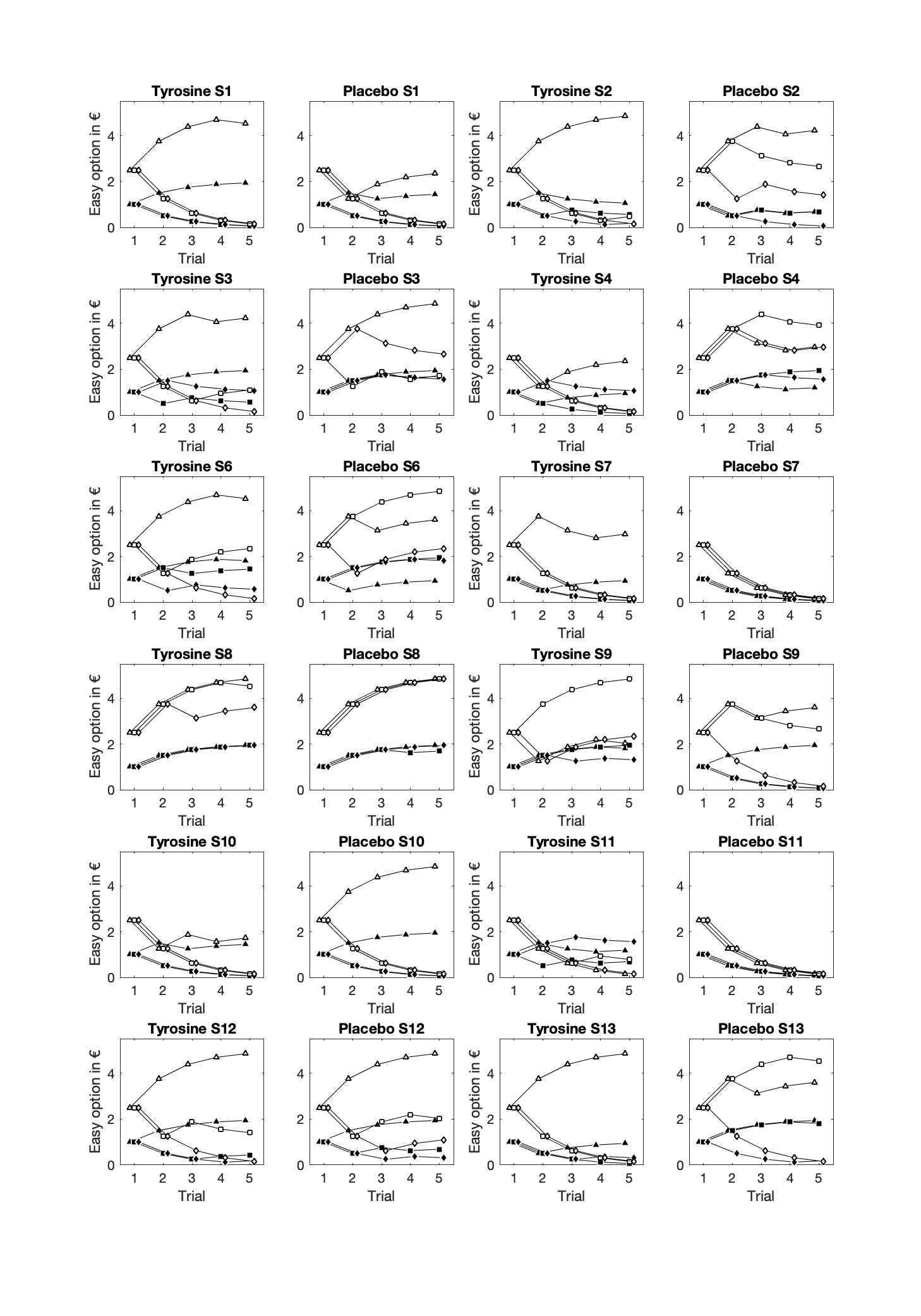

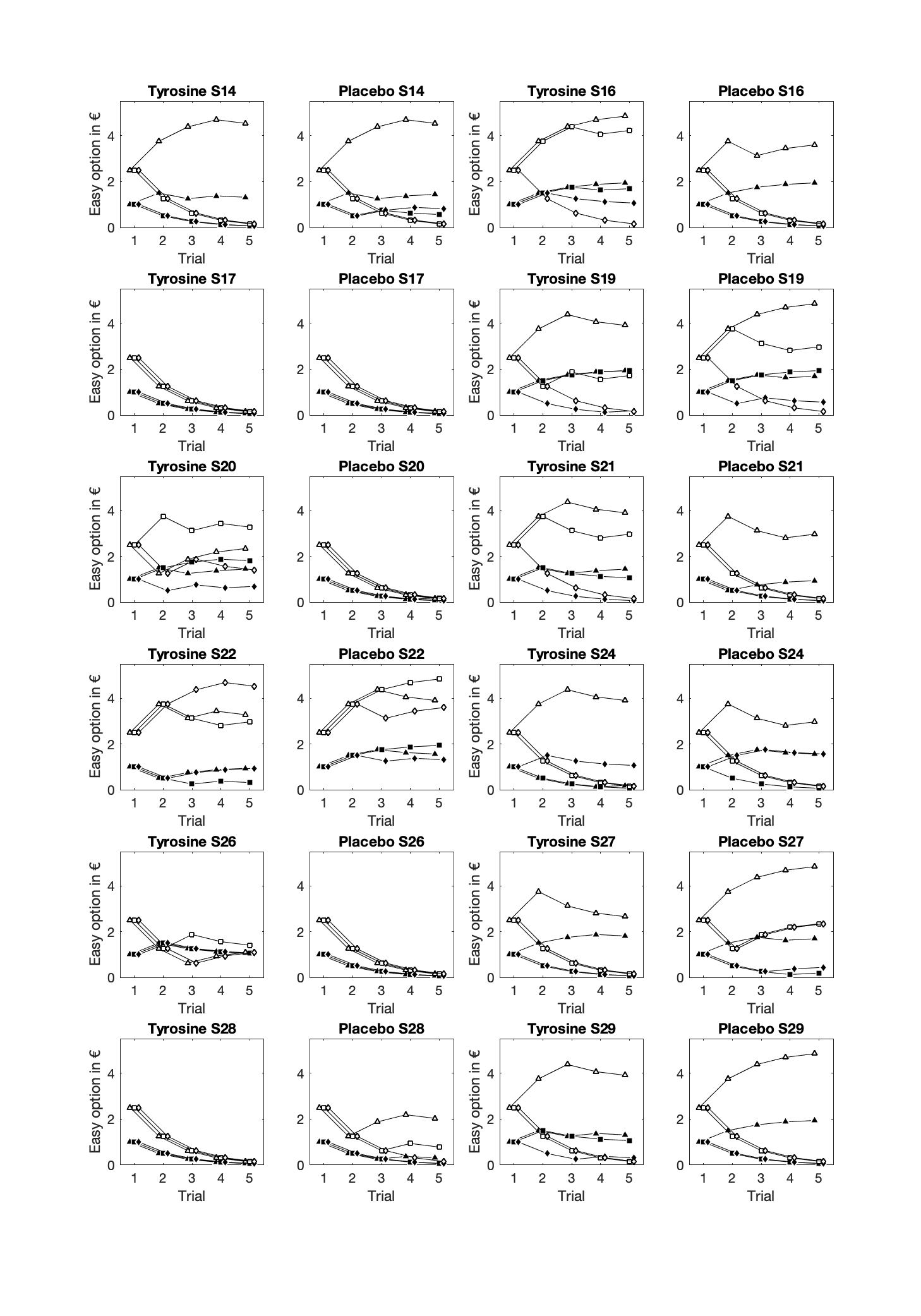

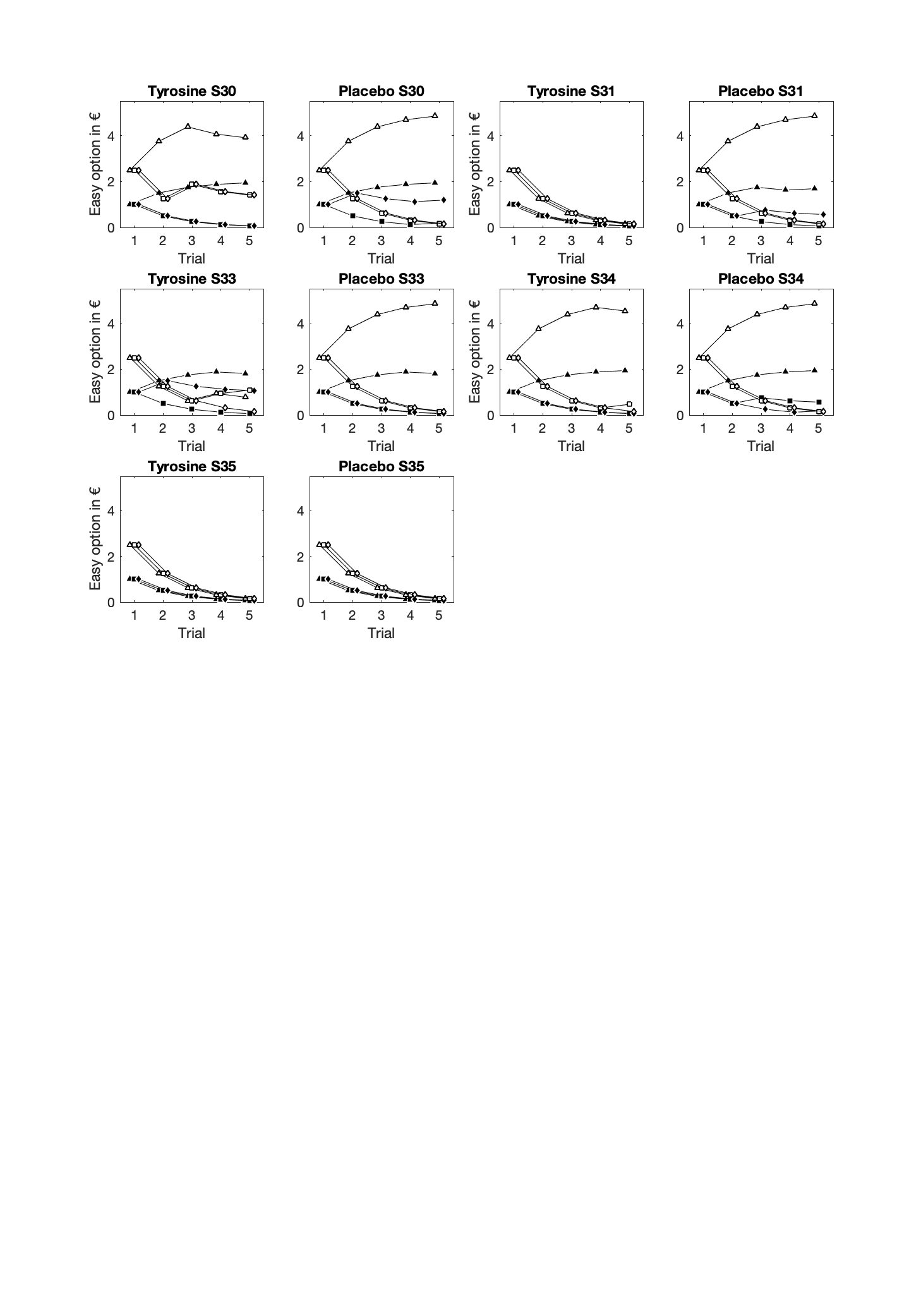

Supplement: S10 File — (DOCX) [file pone.0229294.s010.docx]
