# Supplementary material for: Catecholaminergic modulation of the cost of cognitive control in healthy older adults
Source: PLoS One. 2020 Feb 21;15(2):e0229294. doi: 10.1371/journal.pone.0229294 (PMC7034873; doi:10.1371/journal.pone.0229294)
Supplement: Attachment — Submitted filename: ResponseToReviewers.pdf [file pone.0229294.s011.pdf]

Revision of [PONE-D-19-19882] – Froböse et al.

Dear Ross Otto,

Thank you for encouraging us to submit a revised version of the manuscript for consideration of publication in PLOS ONE.

We would also like to thank the reviewers for the time they spent reviewing our manuscript. They have offered constructive ways to further improve and clarify the manuscript. We took each of the concerns seriously and addressed them in two ways: First, we responded to each reviewer point and highlighted our actions following the reviewer comments (Response to Reviewers, see below). Secondly, we made changes in the manuscript, supplemental material and figure 1 to correct the shortcomings that the reviewers perceived. These edits are pasted below the corresponding reviewer point (italic indent) and adapted in the according documents (see Revised Manuscript and Revised Supplements with tracked changes, changes underlined; Fig1_schematic_V2). Lastly, we provide an unmarked version of the revised manuscript and supplements (see Revised Manuscript and Revised Supplements).

We believe that the changes have considerably improved our paper, and we hope you agree.

We are looking forward to hearing from you.

Yours faithfully,
Monja Froböse, Andrew Westbrook, Mirjam Bloemendaal, Esther Aarts, Roshan Cools

==
ORIGINAL REVIEWS with our responses printed in italic
==

Review Comments to the Author

Reviewer #1: Froböse and colleagues present a Psychopharmacology study aimed at examining whether tyrosine modulates the willingness to exert cognitive effort in older adults. They use a well established previous task, in which effort is putatively manipulated with different levels of difficulty in an N-back task and participants are offered different magnitudes of monetary reward. They test older adults on this task in a double-blind, within-subject, placebo-matched crossover design and test whether the "SV" of exerting cognitive control (as measured using a decision-making task) is modulated by tyrosine. They find that Tyrosine does not modulate the willingness to exert effort per se, but in an exploratory analysis, show that there is an effect of the drug on the willingness to exert effort, but only as a function of trait impulsivity. They interpret their results as trait impulsivity being a marker of dopaminergic efficacy and tyrosine having different effects on motivation depending on levels of impulsivity.

This is a nice study, with appropriate measures, results that will be of interest to the field and atypically, I do not have many issues to raise with the analyses. I would also like to note that it is a nicely transparent study in terms of hypothesised vs exploratory results. My only real concern is about whether this study is actually finding results relating to "effort" or whether all of the results might be interpreted as a change in risk/impulsive preferences.

The authors find that subjective value changes on Tyrosine vs placebo but only as a function of trait impulsivity. The interpretation is that this is a shift in the 'cost' of effort. However, given the nature of the cognitive control task, it is also plausible that this shift is actually more related to differences in risk preference, which may be at least as consistent with the current interpretation. Much of the literature they discuss suggests that D2/D3 in the dopamine system are linked to effort sensitivity, but the same system also regulates impulsivity and risk preferences (Buckholtz et al., 2010, Science). Moreover, there are several aspects of the data that suggest that effort may not be the cost that changes. First, the results show that impulsivity moderates the effect of Tyrosine, but, unless I missed it, 'Need for cognition' – which they label as an effort questionnaire – did not show a similar effect. Second, the N-back task has inevitably high error rates at higher levels of N and this perception of risk may have been what changes rather than the perception of effort. Thus, participants may have felt that there was a greater risk of error, or a greater risk of not getting the monetary reward, rather than cognitive control requiring more effort. Lastly, the confounding of effort and risk might be an alternative explanation of why other studies have shown an effect of dopaminergic manipulations on physical (Le heron et al., 2018; Brain) and cognitive effort (McGuigan et al., 2019, Brain) independently of trait measures, in tasks where the probability of getting rewarded is very high and constant across levels of effort. I therefore feel that the authors should discuss this alternative interpretation at length and also tone down interpretations of the effects being related to effort.

> *We agree that our account that tyrosine effects on subjective value likely reflect changes in the effort cost is speculative. As the reviewer correctly states, one might argue that the impulsivity-dependent effect of tyrosine on the subjective value might be mediated partly by (i) changes in risk avoidance, because more difficult (i.e. higher effort) levels are associated with greater error rates and/or by (ii) changes in impulsivity/reward-sensitivity, leading to an altered emphasis on the benefits (i.e. monetary reward) irrespective of changes in effort costs. The current design does not allow us to entirely dissociate these accounts because reward magnitude and effort levels were not manipulated independently and because we did not control for accuracy differences between the different effort levels (as done for example in* McGuigan et al., 2019*).*

Beyond the practical difficulties of dissociating risk of errors and effort levels, there might also be the fundamental conceptual overlap which may, alternately, help us understand the nature of subjective effort. Namely, it is possible that the reason we experience the feeling of effort in the first place is as a partial warning signal that we are likely to make an error.
We have now toned down the conclusion, emphasizing the limitation that a change in subjective value might reflect changes in perceived effort costs, changes in reward sensitivity (i.e. impulsive choice tendencies), changes in risk avoidance or a combination of the above.

However, note that the following 3 observations render these alternative accounts less plausible.

First, when we take into account tyrosine-induced changes in task-performance (i.e. d') in the choice analysis, the impulsivity-dependent changes in subjective value are still observed (drug x level x impulsivity: F(1, 19) = 4.8, p = 0.041)). This suggests that full indirect modulation via failure (i.e. error) avoidance is unlikely.

Second, participants were informed that payment was not contingent on their task performance. We instructed participants explicitly that they would receive the monetary reward in exchange for the completion of the re-do block if their performance resembled that of the effort execution phase of the same day. Thus, similar to the work led by Le Heron et al., 2018 *and* McGuigan et al., 2019, *the probability of getting rewarded was quite high and comparable across levels of effort, because here the reference point was also based on their personal effort level-dependent performance score. This procedure should at least reduce the frequency of low-effort choices due to risk-/ error-avoidance.*

Third, there was also no evidence for tyrosine-induced changes in participants' impulsive responding in terms of faster response times during decision-making. We do not observe any impulsivity-dependent changes in mean choice RTs (Drug x BIS impulsivity: F(1, 26) = 0.05, p = 0.827; Drug x BIS impulsivity x offer amount: F(1, 26) = 0.11, p = 0.747), although there were some response time changes in the effort execution N-back phase: Tyrosine enhanced the speed of difficult task performance in more impulsive participants, while also reducing their subjective value of difficult task performance. If motor impulsivity would confound subjective value estimation by effort-independent high offer selection, these (trait impulsive) individuals should have higher, instead of lower, subjective values and no parametric modulation of subjective values by effort level. Thus, the combination of response invigoration (perhaps indicating an increase in motor impulsivity) and lower subjective values (i.e. greater avoidance of monetary reward /effort levels) would speak against the interpretation that subjective value changes were mediated by (effort-unrelated) changes in motor impulsivity/reward sensitivity.

To acknowledge these points, we adapted the discussion in the following way:

p. 33, line 676:
"Although we remain puzzled by the fact that we observe any modulation of the effort execution phase by tyrosine that early after administration, <u>the lack of changes in task performance (i.e. d') and</u> the direction of response-time effects render a performance failure account <u>unlikely</u>. Self-report ratings of perceived effort conducted right after effort execution support this interpretation, as ratings did not show (impulsivity-dependent) drug effects.
<u>Moreover, the combination of response invigoration (perhaps indicating an increase in motor impulsivity) and lower subjective values (i.e. greater avoidance of monetary reward/effort levels) would speak against the interpretation that subjective value changes were mediated by (effort-unrelated) changes in motor impulsivity/reward sensitivity</u>."

p. 36, line 751:
<u>"Lastly, the cognitive effort discounting paradigm we employed has several limitations. First, note that subjective values were extracted from participants' choices based on offers</u>

manipulating both the monetary reward and effort level. Thus, intervention effects specific to effort cost and reward benefit sensitivity are not entirely dissociable in the current design. Second, we cannot fully exclude the alternative account that the effect of tyrosine on the subjective value of cognitive effort reflects an effect on risk aversion, given that the higher effort options were also associated with lower accuracy."

In a slight contradiction to my previous point, the authors might want to highlight that there is some evidence in humans that the valuation of cognitive and physical effort occurs in not entirely overlapping systems in the brain (Chong et al., 2017, PLoS Bio). Moreover, in that study the same participants valued cognitive and physical effort differently, both mathematically, and also with only a very weak correlation between how motivated people were to exert effort one domain or the other. This would support the idea that cognitive effort may rely on distinct mechansims.

We acknowledge this point and now provide a more balanced view on the generalizability of physical effort findings to the domain of cognitive effort. We adapted the following points:

p. 4, line 45:
"However, unlike for physical effort, the role of catecholamines is less clear for decision making about cognitive effort and it is still under study whether findings regarding physical effort valuation (entirely) generalize to the cognitive domain (e.g. Chong et al., 2017; Cocker et al., 2012; Hosking et al., 2015; Schmidt et al., 2012).

p. 31, line 637:
"[Moreover, recent work has shown that increased dopamine might promote not just physical effort, but also cognitive control, by offsetting effort costs (Manohar et al., 2015; McGuigan et al., 2019; but see e.g. Chong et al., 2017; Hosking et al., 2015; Schmidt et al., 2012 for domain-general and -specific effects of effort valuation)."

The discussion of the effects being related to "opportunity costs" comes across as a little speculative. However, I think there is additional evidence that the authors could cite to support their claims, specifically Bierholm et al., (2013, Neuropsychopharmacology) and Le Heron et al., (2019, Bioarxiv).

Thank you for the suggestions. We extended the 'opportunity cost' account by further literature emphasizing that dopaminergic medication has been shown to modulate the strength of opportunity cost effects.

p. 35, line 724:
In short, dopamine tone has been proposed to convey local environmental richness, and therefore the opportunity costs of 'sloth' (Beierholm et al., 2013; Niv et al., 2007).

p. 35, line 727:
Indeed, strategic adjustments in the degree to which people perform fast and accurately on cognitive control tasks have been shown to depend on fluctuations in the average reward rate (Otto & Daw, 2019) and the strength of behavioral modulation by (background) average reward-rate was sensitive to pharmacological manipulations of the dopamine system (Beierholm et al., 2013; Le Heron et al., 2019).

Reviewer #2: This manuscript describes a study where the impact of tyrosine, a catecholamine precursor, was investigated on a paradigm that measures the subjective value of control. Specifically, a group of 29 elderly subjects (aged 60 to 75) underwent three sessions of which the last two contrasted the effects of tyrosine intake versus placebo on different levels of the n-back task as well as a subsequent valuation task. The authors did not find support for their main hypothesis, but observed an interesting interaction with Barratt Impulsiveness Scale-scores in exploratory follow-up analyses. The study is interesting, the method is sound, the analyses are well-introduced, and the results clearly discussed. I have no major concerns, but do have five questions / suggestions:

I have a question regarding the general paradigm, and wonder to which extent it might not be a limitation of this study. I was a bit surprised to see how much of an effect the first choice(s) has on the SV: "the magnitude of amount adjustments was cut in half after each adjustment". I understand why this magnitude should reduce over the course of this task, but by making it 50% there is no option to correct a previous mistake. For example, if I was in doubt and accidentally gave the wrong response on the first trial, there is no possibility to reach my real SV over the remainder of this task? This makes the experiment quite sensitive to response errors/noise. Perhaps there are other reasons for this design feature that I am currently not seeing, but I was wondering if the authors could either briefly motivate this, or discuss this limitation (if they agree it is one). Maybe this is also something that could be checked or controlled for empirically (e.g., a participant that shows a pattern where all choices are opposite to their first could suggest something like this).

We agree that the large trial-to-trial adaptation can be considered a weakness because early choices have a much larger impact on the subsequent offer amount than later choices. In addition, every choice is sampled only once, which does not account for the fact that choices are probabilistic. Thus, as the reviewer states, this procedure does not allow complete correction for initial mistakes and might be sensitive to response noise. We have now clarified this in the methods section on page 18 and in the limitation section of the discussion on page 36 (see details below).

*Yet, there were several reasons for including the paradigm in its original form in this study. First, the paradigm has been extensively piloted and used in previous studies (e.g. Chang et al., 2019; Culbreth et al., 2016; Westbrook et al., 2013, 2019), thus we considered it a robust and validated paradigm sensitive to age-related changes in cognitive motivation and effort-manipulations (Westbrook et al., 2013). Second, the staircase procedure makes it a very efficient design, approximating indifference points within 5 choices/repetitions per cell (30 choices: 5 easy-offer adjustments*3 effort levels*2 high-offer amounts). The fact that each level-specific subjective value is based on two independent runs that probe two different maximal offer amounts for the harder options (€2 versus €5) might reduce the impact of initial choice "mistakes" in one of the runs. Moreover, the subjective values as assessed in the low and high offer amount runs show a strong correlation (r = 0.82), which points towards decent test-retest reliability suggesting that the mistakes are infrequent enough to have little influence. Third, from a previous study it seems that the applied titration procedure approximates an indifference point quite reliably (Westbrook et al., 2019). In that study, the staircase procedure as employed here was used to calibrate individual indifference points in order to adjust subsequent choice difficulty (in the fMRI). Indeed, in line with earlier work, participants slowed down for choices close to their pre-defined indifference points. Moreover, choices reliably depended on the pre-determined indifference point: choices evidenced a preference for high-effort choices on high-effort biased trials and for low-effort choices on low-effort biased trials (for details, see Westbrook et al., 2019, p.3939).*

Nevertheless, we did revisit the data following the reviewer's suggestion, and browsed through choice trajectories of individual participants and the average choice trajectory. We pasted the figures below in the appendix of this document and added them to the Supplemental Materials 10. Indeed, participants occasionally show such "corrections" after initial mistakes. For example, S1 placebo session: based on the choice on trial 1, the offer in the high-amount

condition of the 2-back task (unfilled triangles) drops to €1.25 while all subsequent choices were in favor of the 2-back task leading to an increase in easy-option offer. S4, S7 and S20 show the same pattern during the tyrosine session (S4 and S20: high amount 2-back task, S7: low amount 2-back task). S27 shows this pattern for the high amount 3- and 4-back task during the placebo session.

Yet it is difficult to distinguish whether these choices were indeed "corrections" of initial mistakes or reflect their reasonable choices. Given the low frequency of this choice pattern, the calibration of subjective values using two independent choice runs (low and high offer amount for the harder task) and the group plot indicating an overall consistent slope (lacking the sign of systematic initial mistakes), we believe that the current subjective values reflect participants' preferences fairly well.

Based on this reviewer point we implemented the following changes:

p. 18, line 340:
"Note that this titration procedure implies that initial choices weight more heavily than later choices due to the decreasing amount of offer adjustments throughout the choice phase. Yet, from a previous study it seems that the applied titration procedure approximates an indifference point quite reliably (Westbrook et al., 2019). Moreover, each level-specific subjective value is based on two independent choice runs (low and high offer amount), which reduces the impact of initial choice "mistakes" in one run on overall subjective value estimation."

p. 36, line 763:
"Fourth, the employed paradigm does not allow participants to indicate preferences in favor of conducting a more effortful N-back level for an equal or lower monetary amount (i.e. cognitive effort seeking despite monetary "loss") and initial choices weight disproportionally more heavily towards the subjective value due to decreasing offer amount adjustments. As such, replication of this effect using an experimental paradigm that takes into account the probabilistic nature of decision-makers and independently manipulates cost and benefits is recommended."

p. 25, line 521:
Supplemental Material 10 shows the titration procedure (i.e. choice-dependent offer adjustments of the low-effort task) for individual and group data, similar to the description in Figure 2B.

p. 7 of Supplemental Materials:
"Supplemental Material 10. Choice trajectories. Choice-dependent offer adjustments of the low-effort task for the group average (top) and individual participants (bottom). The x-axis reflects the 5 trials of the titration procedure per cell (repeated for 3 effort levels indicated by shapes and 2 amount conditions indicated by the filling). The y-axis shows the offer amounts that can be gained when choosing the easy task. Based on participants' choices, offers for the easy task (always N-back level 1) were adjusted. Note that the amount offered for the high-effort levels were not adjusted but fixed at €5 in the high amount condition (shapes unfilled) and at €2 in the low amount condition (shapes filled). The 3 effort-levels are presented by the according symbols (as in Figure 2C): triangle for the 2-back task, square for the 3-back task and diamond for the 4-back task.
Group figure: Whiskers depict standard errors of the means. For the 2-back task (triangle), the amount offered for the easy task increases as a function of trials because, on average, participants have a preference for the high reward/high effort option. For the 3- and 4-back task (squares and diamonds), the amount offered for the easy task decreases over time because particpants on average have a preference for the easy options.

> *Note that all symbols should be located at x = 1:5. For visualization purposes reducing the overlap in symbols, these are slightly shifted on the x-axis.*
> *In sum, given the low frequency of correction for initial mistakes, the calibration of subjective values using two independent choice runs (low and high offer amount for the harder task) and the group plot indicating an overall consistent slope (lacking the sign of systematic initial mistakes), we believe that the current subjective values reflect participants' preferences fairly well."*

There is a bit of a delay between the N-back task and the effort discounting task (i.e., 3 hours: a break of 90 min, and an fmri session of 90 min). Was this also the case in previous versions of this paradigm, and is it not possible that some participants forgot the relation between some of the shapes and the different levels of difficulty? They are questioned about their experience, but only immediately after the n-back task. Perhaps, not their SV, but their memory of these different levels of difficulty is is further mediated by tyrosine and impulsivity?

> *This aspect indeed differs from previous versions. To expose participants to the shape-effort associations, they practiced the N-back task extensively during the screening session on a separate day (192 trials, 48 per N-back level), and again on the same day of tyrosine/placebo intake (128 trials, 32 per N-back level). In addition, to further reduce the working memory burden, we presented the shape-effort associations (the shapes in order from low to high effort) on a sheet of paper, below the screen that displayed the choice task (p. 17, line 333: "participants also had access to a paper sheet reminding them of the relevant shape-level associations"). The large effect of effort level on subjective values points towards an accurate representation of effort levels. Further, in the larger study we administered a digit span test to probe tyrosine-induced changes in working memory. There were no effects of tyrosine on the digit span score (Table 1: $t(27) = 1.5$, $p = 0.145$) and across participants, changes in digit span scores did not correlate with impulsivity scores ($r = 0.06$, $p = 0.766$). Nevertheless, we cannot exclude that tyrosine (partly) altered the strength of the shape-effort associations as a function of impulsivity. To address this issue, we added the following sentence to the limitations section:*
>
> *p. 36, line 757:*
> *"Third, due to a profound delay of more than 2.5 hours between effort execution and choice phase, we cannot fully exclude the contribution of tyrosine effects on the memory of effort-level representations. Participants were exposed to the shape-effort associations multiple times during practice, a sheet of paper reminded them of the association during the choice task and we observed linear effects of effort-levels on subjective values, but the delay between effort execution and choices was larger here than in previous versions of this paradigm."*

The authors are correct in discussing the interaction with impulsivity scores with caution, given that it is not strong and comes from a set of secondary, exploratory analyses. However, this is currently not acknowledged in the abstract. For example, the authors could mention that: "Instead, in line with our previous study, *exploratory analyses showed that* drug effects varied...".

> *We now acknowledge in the abstract the exploratory nature of the impulsivity-dependent finding.*
>
> *p. 2 line 9: "Instead, in line with our previous study, exploratory analyses indicated that drug effects varied as a function of participants' trait impulsivity scores."*

It is mentioned that participants ate a very similar tasting banana yoghurt in the placebo condition. However, participants might not have tasted the difference, but still experienced a different sensation later during the session. Therefore, was awareness also tested, or does there exist other data on this from previous studies?

There were very little tyrosine effects beyond the primary behavioral measures. For example, blood pressure, heart rate and urine metabolites were mainly un-affected (Table 1). Also, subjective mood ratings did not change after tyrosine administration (Table 1). To probe awareness, participants reported after the second testing day their belief about the order of placebo and tyrosine sessions. These data are available for 23 out of 29 participants. Out of 23 participants, 7 guessed the order of manipulation correctly, 10 guessed incorrectly and 6 refrained from giving any judgement because they did not know. Thus, the majority of these participants were unaware of the true intervention order. We added these scores to the results section (see below).

*It is difficult to evaluate how these scores relate to previous work in healthy adults due to variability in tyrosine doses and the lack of reporting on awareness of intervention. The absence of mood changes is in line with recent other studies also conducted in healthy young adults without any other interventions (e.g. stress or exercise), in which however lower doses of tyrosine were administered (2.0 g: Colzato et al., 2013, 2014; Steenbergen et al., 2015; 0.5 g: Leathwood & Pollet, 1983). Most studies report that blood pressure and heart rate were not affected by the intervention (Colzato et al., 2013, 2014; Steenbergen et al., 2015; but Thomas et al., 1999: *increase* during working memory after 150 mg/kg; Sved et al., 1979: *decrease* after 50 and 200 mg/kg, but in rats). In sum, unlike other catecholaminergic challenges, such as methylphenidate (e.g. Froböse et al., 2018), tyrosine does not seem to affect subjective and physiological measures.*

p. 10, line 206:
"To probe awareness of the drug manipulation, participants reported after the second testing day their belief about the order of placebo and tyrosine sessions."

p. 29, line 599:
"3.3 Self-report N-back questionnaire and awareness of intervention"

p. 30, line 614:
"After the second experimental session, participants reported their belief about the order of placebo and tyrosine sessions. Data are available for 23 out of 29 participants. 7 out of 23 participants judged the order correctly, 10 judged the order incorrectly and 6 refrained from giving any judgement because of a lack of confidence. Thus, the majority of these participants were unaware of the true intervention order."

Figure 1 does not describe/depict the three paradigms (but the reference to this figure in the method section on page 10 suggests it does), or the 90 min break. Also, the lower timeline is a bit confusing as to whether it is depicting time (i.e., 20 min, 45 min, 189 min) relative to T0, or relative to the previous time point (i.e., 45 min is 45 + 20 min later than T0, etc.)?

Sorry for the confusion and thank you for raising this. We adapted figure 1 and hope that it now clarifies these points. For clarification purposes, we now moved the information on peak levels of tyrosine plasma levels to the figure caption.

p. 11, line 216:
Plasma levels of tyrosine have been shown to peak 90-120 minutes after the oral tyrosine administration.

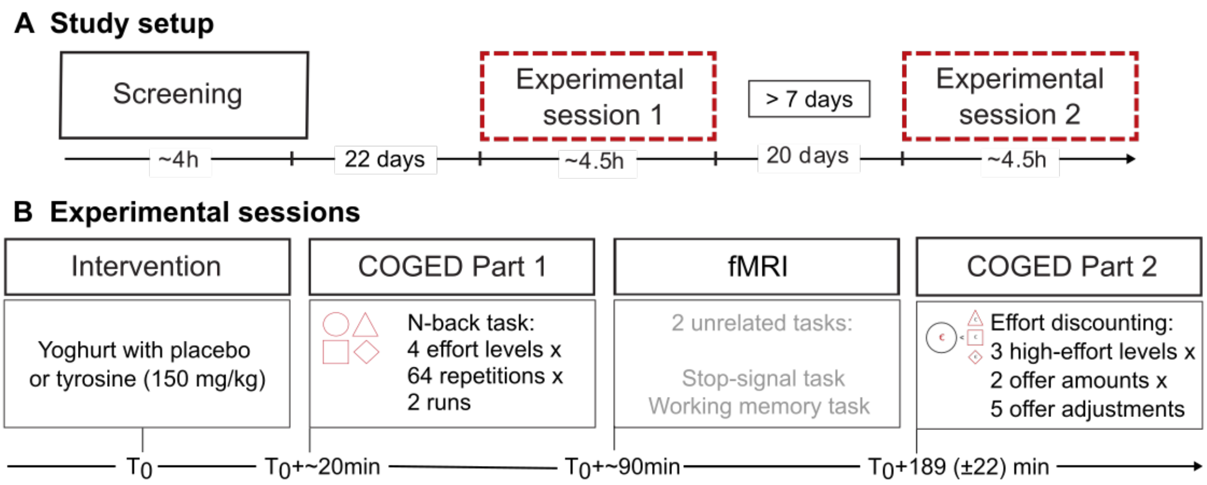

Signed,

Senne Braem

APPENDIX

Supplemental Material 10. Choice trajectories. *Choice-dependent offer adjustments of the low-effort task for the group average (top) and individual participants (bottom). The x-axis reflects the 5 trials of the titration procedure per cell (repeated for 3 effort levels indicated by shapes and 2 amount conditions indicated by the filling). The y-axis shows the offer amounts that can be gained when choosing the easy task. Based on participants' choices, offers for the easy task (always N-back level 1) were adjusted. Note that the amount offered for the high-effort levels were not adjusted but fixed at €5 in the high amount condition (shapes unfilled) and at €2 in the low amount condition (shapes filled). The 3 effort-levels are presented by the according shapes (as in Figure 2C): triangle for the 2-back task, square for the 3-back task and diamond for the 4-back task.*
Group figure: Whiskers depict standard errors of the means. For the 2-back task (triangle), the amount offered for the easy task increases as a function of trials because, on average, participants have a preference for the high reward/high effort option. For the 3- and 4-back task (squares and diamonds), the amount offered for the easy task decreases over time because particpants on average have a preference for the easy options.
Note that all shapes should be located at x = 1:5. For visualization purposes reducing the overlap in symbols, these are slightly shifted on the x-axis.
In sum, given the low frequency of correction for initial mistakes, the calibration of subjective values using two independent choice runs (low and high offer amount for the harder task) and the group plot indicating an overall consistent slope (lacking the sign of systematic initial mistakes), we believe that the current subjective values reflect participants' preferences fairly well.

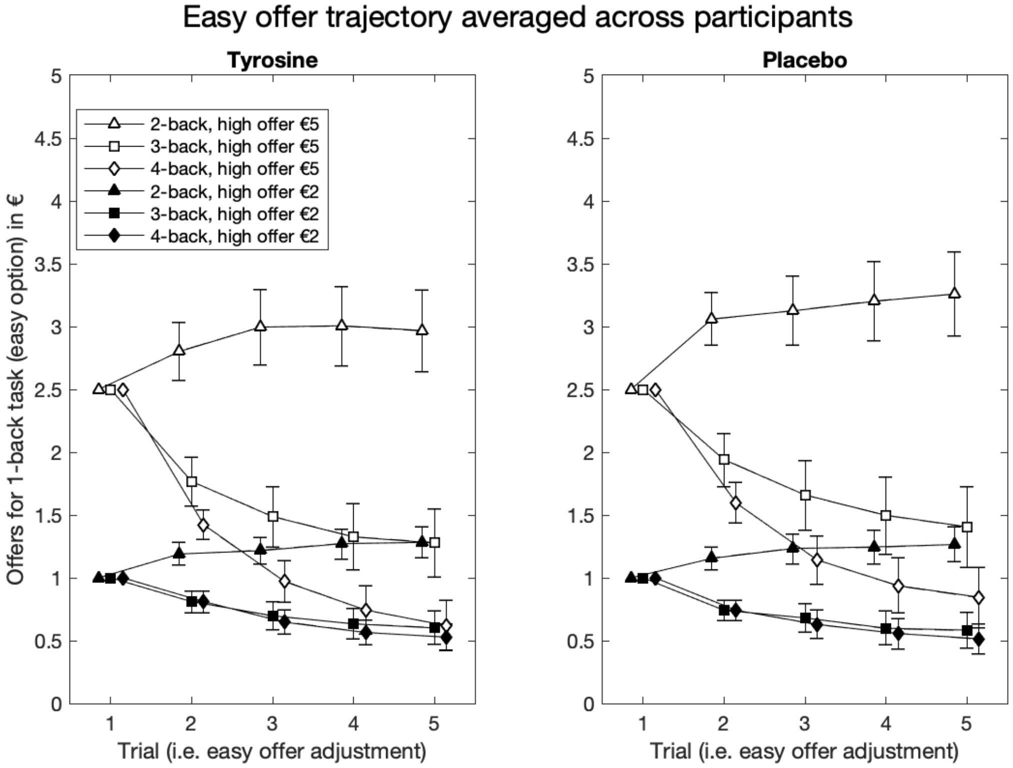

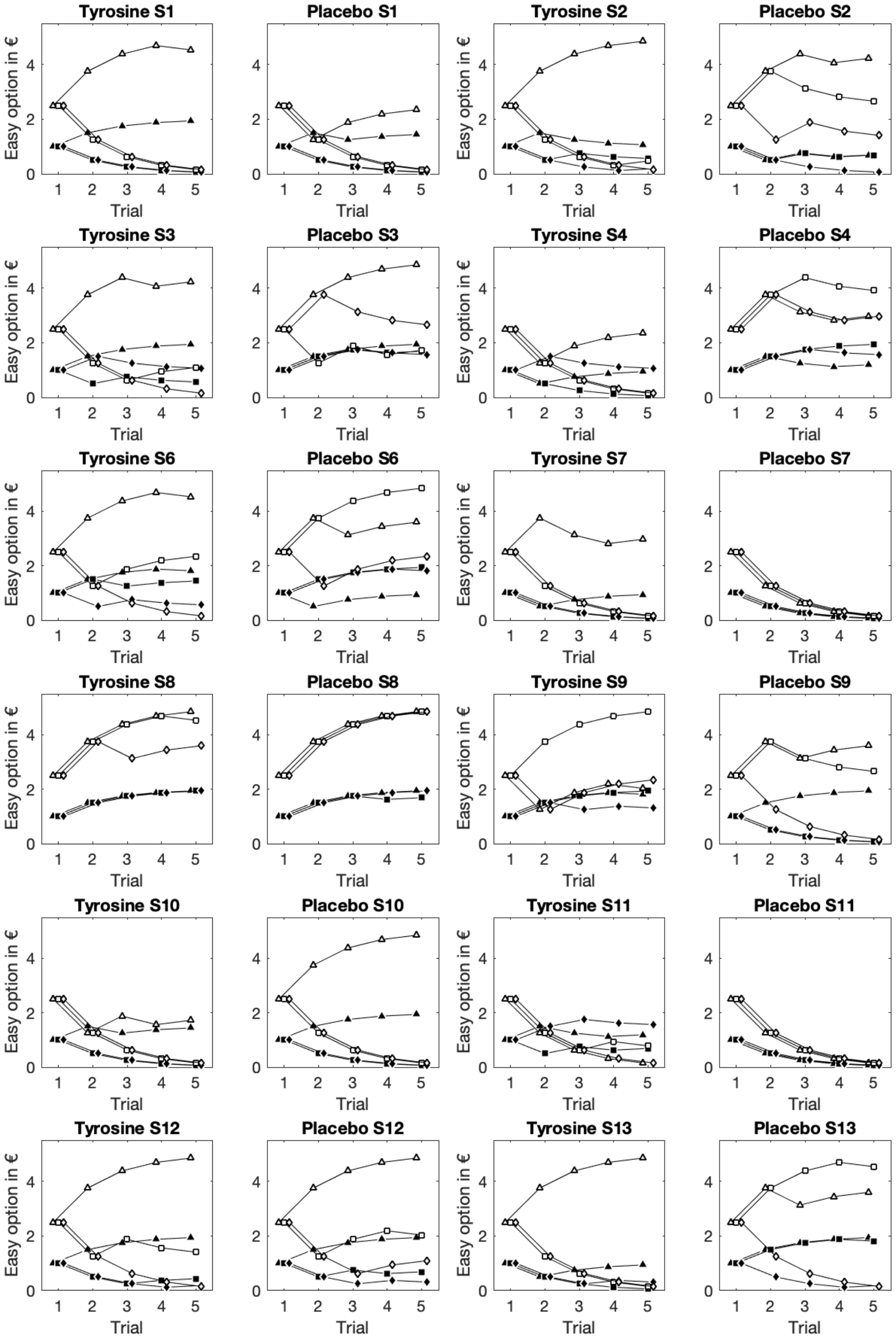
Tyrosine S1
Placebo S1
Tyrosine S2
Placebo S2
Tyrosine S3
Placebo S3
Tyrosine S4
Placebo S4
Tyrosine S6
Placebo S6
Tyrosine S7
Placebo S7
Tyrosine S8
Placebo S8
Tyrosine S9
Placebo S9
Tyrosine S10
Placebo S10
Tyrosine S11
Placebo S11
Tyrosine S12
Placebo S12
Tyrosine S13
Placebo S13
Easy option in €
Trial

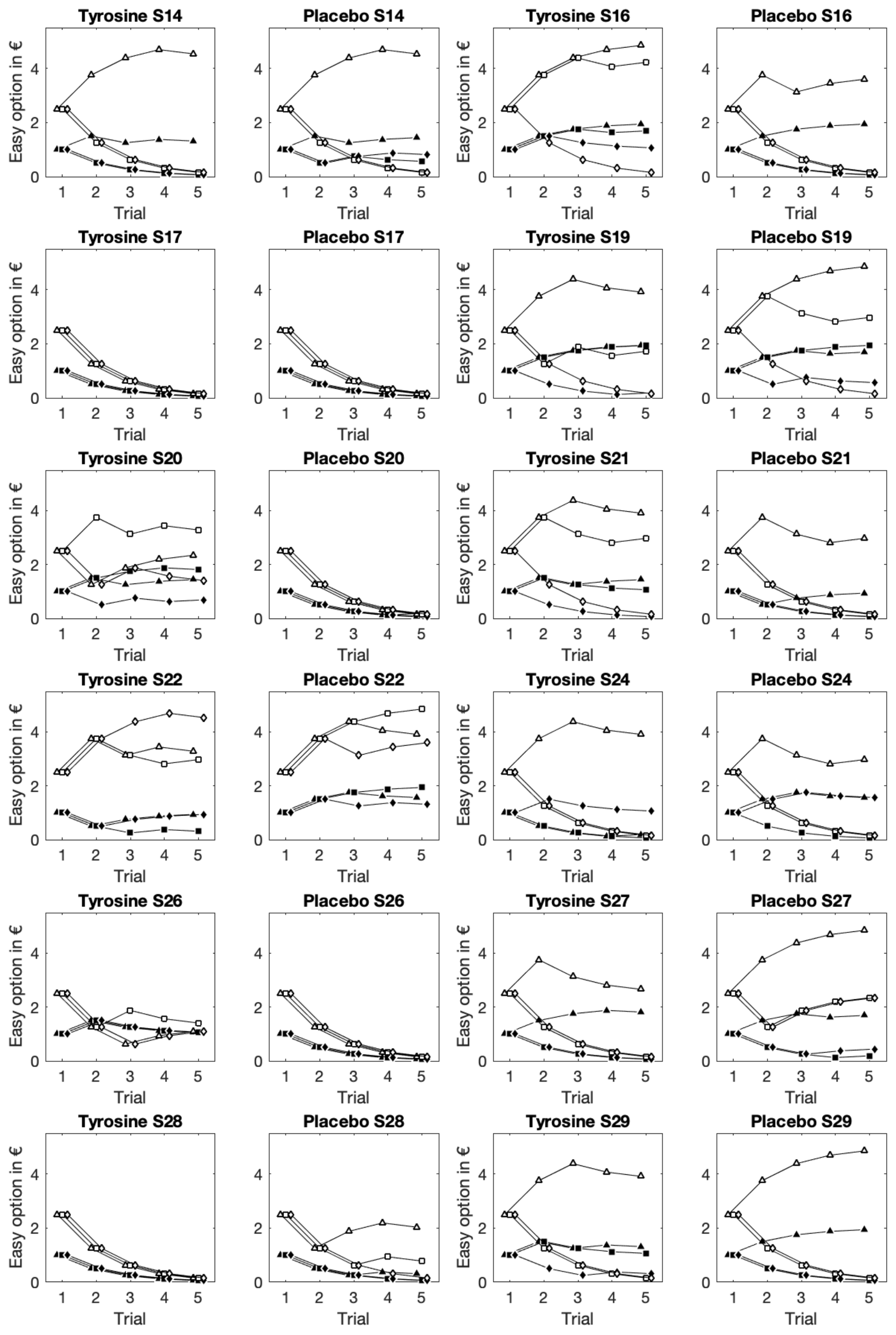
Tyrosine S14
Placebo S14
Tyrosine S16
Placebo S16
Tyrosine S17
Placebo S17
Tyrosine S19
Placebo S19
Tyrosine S20
Placebo S20
Tyrosine S21
Placebo S21
Tyrosine S22
Placebo S22
Tyrosine S24
Placebo S24
Tyrosine S26
Placebo S26
Tyrosine S27
Placebo S27
Tyrosine S28
Placebo S28
Tyrosine S29
Placebo S29
Easy option in €
Trial

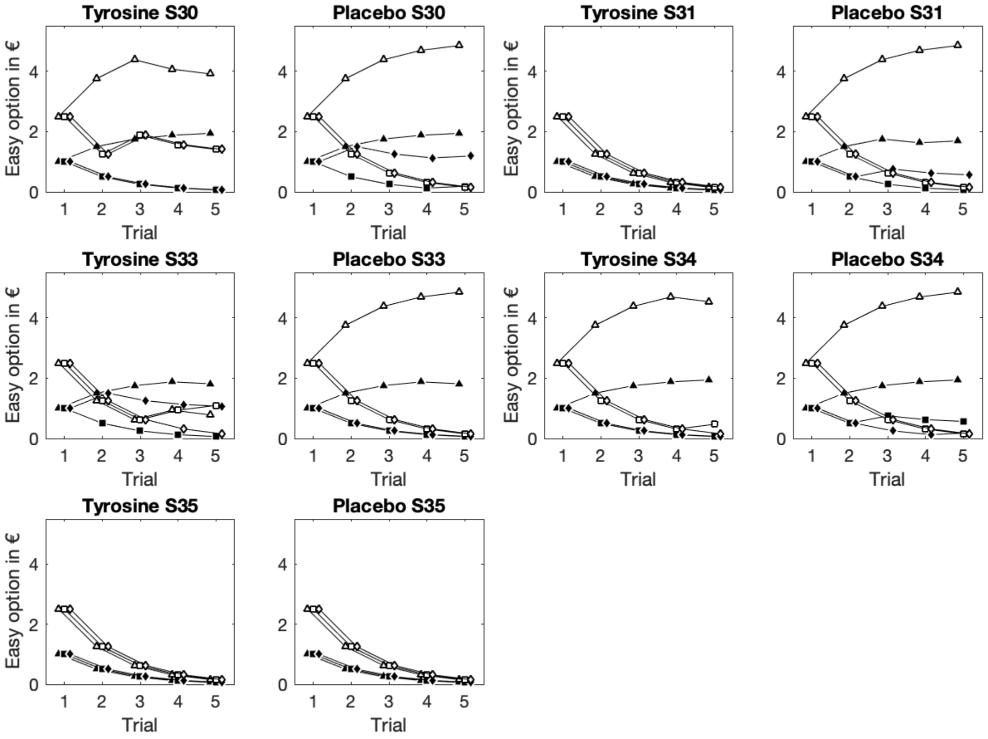
Tyrosine S30
Placebo S30
Tyrosine S31
Placebo S31
Tyrosine S33
Placebo S33
Tyrosine S34
Placebo S34
Tyrosine S35
Placebo S35
Easy option in €
Trial

REFERENCES

Beierholm, U., Guitart-Masip, M., Economides, M., Chowdhury, R., Düzel, E., Dolan, R., & Dayan, P. (2013). Dopamine Modulates Reward-Related Vigor. *Neuropsychopharmacology*, *38*(8), 1495–1503. https://doi.org/10.1038/npp.2013.48

Chang, W. C., Westbrook, A., Strauss, G. P., Chu, A. O. K., Chong, C. S. Y., Siu, C. M. W., Chan, S. K. W., Lee, E. H. M., Hui, C. L. M., Suen, Y. M., Lo, T. L., & Chen, E. Y. H. (2019). Abnormal cognitive effort allocation and its association with amotivation in first-episode psychosis. *Psychological Medicine*, 1–11. https://doi.org/10.1017/s0033291719002769

Chong, T. T.-J., Apps, M., Giehl, K., Sillence, A., Grima, L. L., & Husain, M. (2017). Neurocomputational mechanisms underlying subjective valuation of effort costs. *PLOS Biology*, *15*(2), e1002598. https://doi.org/10.1371/journal.pbio.1002598

Cocker, P. J., Hosking, J. G., Benoit, J., & Winstanley, C. A. (2012). Sensitivity to cognitive effort mediates psychostimulant effects on a novel rodent cost/benefit decision-making task. *Neuropsychopharmacology*, *37*(8), 1825–1837. https://doi.org/10.1038/npp.2012.30

Colzato, L. S., Jongkees, B. J., Sellaro, R., & Hommel, B. (2013). Working memory reloaded: tyrosine repletes updating in the N -back task. *Frontiers in Behavioral Neuroscience*, *7*, 1–5. https://doi.org/10.3389/fnbeh.2013.00200

Colzato, L. S., Jongkees, B. J., Sellaro, R., van den Wildenberg, W. P. M., & Hommel, B. (2014). Eating to stop: Tyrosine supplementation enhances inhibitory control but not response execution. *Neuropsychologia*, *62*, 398–402. https://doi.org/10.1016/j.neuropsychologia.2013.12.027

Culbreth, A., Westbrook, A., & Barch, D. (2016). Negative Symptoms are Associated with an Increased Subjective Cost of Cognitive Effort. *Journal of Abnormal Psychology*, *125*(4), 528–536. https://doi.org/10.1016/j.physbeh.2017.03.040

Froböse, M. I., Swart, J. C., Cook, J. L., Geurts, D. E. M., den Ouden, H. E. M., & Cools, R. (2018). Catecholaminergic modulation of the avoidance of cognitive control. *Journal of Experimental Psychology: General*, *147*(12), 1763–1781. https://doi.org/10.1037/xge0000523

Hosking, J. G., Floresco, S. B., & Winstanley, C. A. (2015). Dopamine Antagonism Decreases Willingness to Expend Physical, But Not Cognitive, Effort: A Comparison of Two Rodent Cost / Benefit Decision-Making Tasks. *Neuropsychopharmacology*, *40*, 1005–1015. https://doi.org/10.1038/npp.2014.285

Le Heron, C., Kolling, N., Plant, O., Kienast, A., Janska, R., Ang, Y.-S., Fallon, S., Husain, M., & Apps, M. A. J. (2019). Dopamine and motivational state drive dynamics of human decision making. *BioRxiv*, 709857. https://doi.org/10.1101/709857

Le Heron, C., Manohar, S., Plant, O., Muhammed, K., Griffanti, L., Nemeth, A., Douaud, G., Markus, H. S., & Husain, M. (2018). Dysfunctional effort-based decision-making underlies apathy in genetic cerebral small vessel disease. *Brain*, *141*(11), 3193–3210. https://doi.org/10.1093/brain/awy257

Leathwood, P. D., & Pollet, P. (1983). Diet-induced mood changes in normal populations. *Journal of Psychiatric Research*, *17*(2), 147–154.

Manohar, S. G., Chong, T. T. J., Apps, M. A. J., Batla, A., Stamelou, M., Jarman, P. R., Bhatia, K. P., & Husain, M. (2015). Reward Pays the Cost of Noise Reduction in Motor and Cognitive Control. *Current Biology*, *25*(13), 1707–1716. https://doi.org/10.1016/j.cub.2015.05.038

McGuigan, S., Zhou, S. H., Brosnan, M. B., Thyagarajan, D., Bellgrove, M. A., & Chong, T. T. J. (2019). Dopamine restores cognitive motivation in Parkinson's disease. *Brain*, *142*(3), 719–732. https://doi.org/10.1093/brain/awy341

Niv, Y., Daw, N. D., Joel, D., & Dayan, P. (2007). Tonic dopamine: Opportunity costs and the control of response vigor. *Psychopharmacology*, *191*(3), 507–520. https://doi.org/10.1007/s00213-006-0502-4

Otto, A. R., & Daw, N. D. (2019). The opportunity cost of time modulates cognitive effort. *Neuropsychologia*, *123*(May 2018), 92–105. https://doi.org/10.1016/j.neuropsychologia.2018.05.006

Schmidt, L., Lebreton, M., Clery-Melin, M.-L., Daunizeau, J., & Pessiglione, M. (2012). Neural Mechanisms Underlying Motivation of Mental Versus Physical Effort. *PLoS Biology*, *10*(2). https://doi.org/10.1371/journal.pbio.1001266

Steenbergen, L., Sellaro, R., Hommel, B., & Colzato, L. S. (2015). Tyrosine promotes cognitive flexibility: Evidence from proactive vs. reactive control during task switching performance. *Neuropsychologia*, *69*, 50–55. https://doi.org/10.1016/j.neuropsychologia.2015.01.022

Sved, A. F., Fernstrom, J. D., & Wurtman, R. J. (1979). Tyrosine administration reduces blood pressure and enhances brain norepinephrine release in spontaneously hypertensive rats. *Proceedings of the National Academy of Sciences U.S.A*, *76*(7), 3511–3514. https://doi.org/10.1073/pnas.76.7.3511

Thomas, J. R., Lockwood, P. A., Singh, A., & Deuster, P. A. (1999). Tyrosine improves working memory in a multitasking environment. *Pharmacology Biochemistry and Behavior*, *64*(3), 495–500. https://doi.org/10.1016/S0091-3057(99)00094-5

Westbrook, A., Kester, D., & Braver, T. S. (2013). What Is the Subjective Cost of Cognitive Effort? Load, Trait, and Aging Effects Revealed by Economic Preference. *PloS One*, *8*(7), 1–8. https://doi.org/10.1371/journal.pone.0068210

Westbrook, A., Lamichhane, B., & Braver, T. (2019). The subjective value of cognitive effort is encoded by a domain-general valuation network. *Journal of Neuroscience*, *39*(20), 3934–3947. https://doi.org/10.1523/JNEUROSCI.3071-18.2019
